# The Impact of *Weizmannia coagulans* BC99 on Anxiety and Depression: An 8-Week Clinical Pilot Study Through the Gut Microbiota–Brain Axis

**DOI:** 10.3390/nu17193087

**Published:** 2025-09-28

**Authors:** Shanshan Tie, Yujia Pan, Chenguang Pang, Azeem Saman, Yao Dong, Shuguang Fang, Jianguo Zhu, Ying Wu, Shaobin Gu

**Affiliations:** 1 College of Food and Bioengineering, Henan University of Science and Technology, Luoyang 471000, China; tieshanshan@haust.edu.cn (S.T.);; 2 Henan Engineering Research Center of Food Material, Henan University of Science and Technology, Luoyang 471023, China; 3 Wecare Probiotics R&D Centers (WPC), Wecare Probiotics Co., Ltd., Suzhou 215200, China; 4 Henan Engineering Research Center of Food Microbiology, Henan University of Science and Technology, Luoyang 471000, China

**Keywords:** probiotic, anxiety, depression, gut microbiota-brain axis, clinical trial

## Abstract

**Background**: An imbalance in the immune system, stress response, and gut microbiota can contribute to the onset and progression of anxiety and depression. This pilot study aimed to explore the effect of *Weizmannia coagulans* BC99 on anxiety and depression through the gut microbiota–brain axis. **Methods**: A total of 79 participants with Hamilton Depression Rating Scale (HAMD-17) ≥ 8 or Hamilton Anxiety Rating Scale (HAMA-14) ≥ 7 were included and completed the study. The participants were randomly assigned to either the placebo group or the BC99 intervention group. The intervention lasted 8 weeks, with participants receiving either dextrin (3 g/day) or BC99 probiotics (3 g/day, 5 × 10^9^ CFU) daily. Pre- and post-intervention comparisons were made on HAMD and HAMA scores, inflammatory cytokines, neurotransmitters, gut microbiota, and short-chain fatty acids. **Results**: Results showed that after 8 weeks, BC99 and placebo intervention were effective in reducing HAMD and HAMA scores, and HAMD and HAMA scores in BC99 group were reduced by 2.40 and 5.53 points compared the placebo group, and the response and remission rates were also higher than that of the placebo, but there was no significant difference. BC99 also could regulate the levels of inflammatory cytokines IL-17 and IL-10, and increase neurotransmitter levels of γ-GABA and NO. Moreover, compared with the placebo group, BC99 also enhanced the abundance of beneficial bacteria such as *Faecalibactrium*, *Megamonas*, *Dialister*, and *Agathobacter*, which were closely associated with clinical indicators of mental disorders, and increased the production of short-chain fatty acids. **Conclusions**: These preliminary findings suggested that BC99 might alleviate anxiety and depression symptoms by regulating the level of neurotransmitters or the change of microbiota, which needed further verification in subsequent animal or clinical experiments.

## 1. Introduction

Anxiety and depression, as common psychological disorders, affect approximately 10% of the global population and cause significant personal and even social burdens. It is estimated that about 85% of people with depression may experience anxiety symptoms, and likewise for up to 90% of people with depressive disorders [1]. The onset of this disease is often accompanied by mental stress, behavioral changes, and, in severe cases, it can also increase the risk of complications such as cancer and suicide [2]. Despite advances in antidepressant therapies, their effectiveness remains suboptimal, with side effects including gastrointestinal issues, weight gain, sexual dysfunction, and other adverse reactions [3,4]. As such, there is an urgent need for novel strategies to intervene in anxiety and depression.

Recent studies have revealed a close relationship between gut microbiota and mood disorders, drawing increasing attention to the role of gut microbial communities in the onset and progression of these mood disorders [5,6]. The gut microbiota is composed of a vast array of bacteria, fungi, and other micro-organisms that inhabit the intestinal tract. Once humans have mood disorders, the composition and function of gut microbiota can change, which is known as dysbiosis [7]. For example, Hao et al. established a classic depression mouse model by the induction of mild pressure, and the mice showed depressive behavior and gut microbiota imbalance [8]. The reduction of gut microbiota diversity could inhibit short-chain fatty acid (SCFA) production, promote the production of intestinal inflammation, and affect neurotransmitter synthesis and metabolism, thereby influencing brain function and exacerbating anxiety and depression symptoms [9]. This highlights the intrinsic connection between the gut microbiota and the brain, known as the gut microbiota–brain axis. This two-way communication, involving the vagus nerve, immune modulation, and microbial metabolism, plays a crucial role in regulating emotions, cognitive function, and behavior. As a result, the gut microbiota–brain axis has emerged as a promising target for interventions in mood disorders [10,11]. Understanding the mechanisms underlying the gut–brain relationship is crucial for developing new therapeutic approaches.

Probiotics, according to the definition of the World Health Organization, are living micro-organisms that have beneficial effects on the host’s health when ingested in sufficient amounts [12]. Probiotics, as an important mediator of gut microbiota regulation, have gradually become a research hotspot in recent years for their potential in mood disorders [13]. Probiotics can improve mental health by mediating multiple pathways, such as regulating the neurotransmitters, metabolites, inflammatory cytokines, and the immune system [14,15]. Animal and clinical research reports can also support the effectiveness of probiotics in improving anxiety and depression. For example, an 8-week multi-strain probiotics adjuvant study showed that, compared with placebo group, the depressive symptoms were improved in the probiotic-intervention group [16]. *Lactobacillus plantarum* JYLP-326 could also relieve anxiety, depression, and insomnia symptoms in college students by regulating gut microbiota and their metabolism [17]. This allows certain probiotics to be viable psychobiotics for alleviating psychological disorders. Psychobiotics are generally living bacteria that can bring mental health benefits to the host by interacting with intestinal commensal bacteria after ingestion, and have the advantages of regulating gut microbiota, producing and transmitting neurotransmitters, etc. [18,19]. Therefore, psychobiotics, as a new, natural, and safe probiotic, may be a good choice for alleviating anxiety and depression.

In recent years, there has been an increasing interest in the *Bacillus* species as probiotics due to their high tolerance to unfavorable factors during processing and storage. *Weizmannia coagulans* BC99, as a spore-forming and lactic acid-producing bacterium, has been generally considered safe by the US Food and Drug Administration (FDA) [20]. BC99 has the potential to optimize intestinal homeostasis, enhance immunity, regulate neurological function and important metabolites, and provide other multidimensional health benefits [12,21]. The previous results in in vitro and animal experiments showed that BC99 could effectively reduce the level of inflammation cytokines and promote the release of neurotransmitters. For example, BC99 could effectively alleviate the cognitive behavior of cognitively impaired mice, regulate the levels of inflammatory cytokines and neurotransmitters in the brain, and promote the production of SCFAs, so as to improve the cognitive impairment of mice [22]. However, the clinical effectiveness of BC99 in relieving anxiety and depression has yet to be fully explored. Therefore, this study conducted a randomized, double-blind, placebo-controlled pilot study to evaluate the intervention effect of BC99 on anxiety and depression via the gut microbiota–brain axis. The changes in the anxiety and depression scale, inflammatory cytokines, neurotransmitters, gut microbiota, SCFAs, and the correlation among related indexes were analyzed. This research aims to provide a theoretical foundation for the clinical application of BC99 in the nutritional intervention of anxiety and depression.

## 2. Materials and Methods

### 2.1. Participants

A randomized, double-blind, placebo-controlled study protocol was conducted in accordance with the World Medical Association Declaration of Helsinki. The study was approved by the Ethics Committee of the First Affiliated Hospital of Henan University of Science and Technology (approval number: 2024-03-K0083) in April 2024. The trial was registered at ClinicalTrials.gov (NCT06629441). The pilot study was conducted in Luoyang, Henan, China, from June to October 2024.

A total of 82 adults were recruited via bulletin boards at the Henan University of Science and Technology and public advertisement in the surrounding communities, mainly graduate students from colleges facing scientific research or employment pressure, or adults with job pressure in different communities. Inclusion criteria included the following: (1) aged 18 to 65 years old; (2) Hamilton Depression Rating Scale (HAMD-17) scores ≥ 8 or Hamilton Anxiety Rating Scale (HAMA-14) scores ≥ 7; (3) Participants voluntarily completed a written and signed informed consent form to participate in this study. Exclusion criteria included the following: (1) individuals with a definite previous diagnosis of other mental disorders, intellectual disability, bipolar disorder, treatment-resistant depression, or suicidal ideation; (2) pregnant or breastfeeding women; (3) individuals with allergies or those abusing alcohol or psychotropic substances; (4) those who had used antibiotics or probiotics affecting gut microbiota for more than 1 week within 1 month prior to the study; and (5) participants who discontinued the study or were unable to participate for any other reasons. All participants received a detailed description of the study protocol and voluntarily signed an informed consent form before participation.

### 2.2. Experimental Design and Intervention

**Figure 1:**
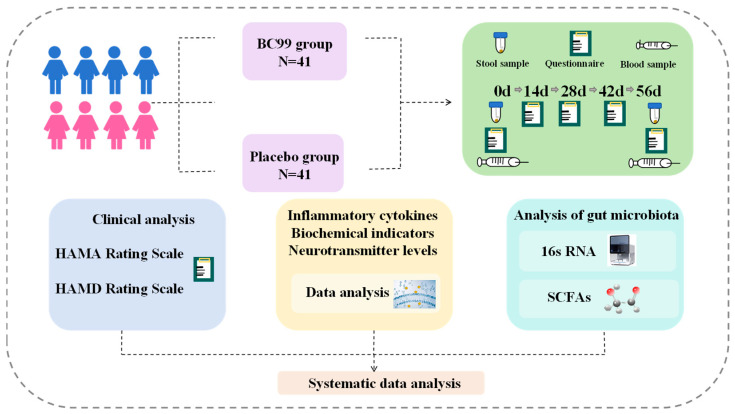
The flow chart of the clinical trial study.

The 82 eligible participants were randomly assigned to the placebo group (*n* = 41) and the BC99 intervention group (*n* = 41). Among them, 79 participants completed the entire study. Participants in the probiotic group received 3 g of BC99 bacterial powder formulation (5 × 10^9^ CFU) daily, while the placebo group received 3 g of dextrin powder without probiotics (Figure 1). The intervention lasted for 8 weeks, during which time participants were asked to record daily product administration, missed doses, and possible adverse symptoms. Researchers could regularly remind participants to take products on time and fill in diaries through WeChat or Message. During the study, participants attended 5 visits on the first and last days (baseline and week 8). At each follow-up visit, researchers also counted the number of remaining bacterial powders in the study. The changes in HAMD-17 and HAMA-14 scores, inflammatory cytokines, biochemical indicators, neurotransmitters, and gut microbiota for blood or stool samples were analyzed. After the intervention, the remaining product and packaging were recovered.

Randomization and blinding. An independent statistician generated a simple randomization sequence in R (v4.3.2) to allocate participants 1:1 to BC99 or placebo (no blocks or stratification). A third party safeguarded the full sequence and prepared sequentially numbered, identical investigational product kits. Investigators enrolled eligible participants and assigned the next available kit number, maintaining allocation concealment. Participants, investigators, clinical staff, outcome assessors, and data analysts remained blinded throughout. BC99 and placebo sachets were matched for appearance, weight, taste, and packaging.

### 2.3. Study Products

The probiotic group received a daily dose of 3 g of BC99 bacterial powder, containing 5 × 10^9^ CFU, produced using a unique microencapsulation technique to ensure the stability and effectiveness of BC99. The placebo group received an identical product composed of dextrin powder. Both BC99 and placebo formulations were manufactured and supplied by Wecare Probiotics Co., Ltd. (Suzhou, China). The packaging for both products was identical, with each bag containing 3 g of product, and both were stored at 4 °C to maintain stability.

### 2.4. Questionnaires

During the experiment, HAMD-17, HAMA-14, Pittsburgh Sleep Quality Index (PSQI), and General Self-efficacy Scale (GSES) were used to understand the anxiety and depression of participants. Scores ≥8 on the HAMD-17 and ≥7 on the HAMA-14 indicated the subjects experienced depression and anxiety, respectively. The detailed grade classification referred to the literature reported by Bonsaksen et al. [23]. PSQI was used to evaluate the sleep quality of the participants before and after participating in the clinical trial. The higher the score, the lower the sleep quality of the participants. Generally speaking, a total score of less than or equal to 5 points indicated that the sleep quality of the participant was good, and a total score of more than 5 points indicated poor sleep quality [24]. GSES took feelings, thoughts, and behaviors of participants as evaluation indicators, including 10 items, each scored from 1 to 4 points, and a total score ranging from 10 to 40 points. The higher the score, the higher the self-efficacy [25].

### 2.5. Clinical Measure

Blood samples were collected before and after 8 weeks of intervention to assess biochemical indicators by clinical standard measurements. After fasting for at least 10 h, blood was collected and centrifuged, and serum was stored at −80 °C for analysis. Serum levels of inflammatory cytokines (interleukin-17 [IL-17], interleukin-10 [IL-10]) and neurotransmitters (gamma-aminobutyric acid [γ-GABA], nitric oxide [NO], cystatin C [Cys-C], and neuropeptide Y [NPY]) were measured using commercial kits from Shanghai Hepeng Biotechnology Co., Ltd. (Shanghai, China).

### 2.6. Collection of Fecal Samples

Fresh fecal samples were collected during fasting and stored at −80 °C for subsequent analysis of gut microbiota and short-chain fatty acids (SCFAs).

### 2.7. Gut Microbiota

The fecal samples from the probiotic group and the placebo group collected above were analyzed for gut microbiota using the 16S rDNA sequencing technique. The sequencing process included DNA extraction and detection, PCR amplification, product purification, library preparation and quality inspection, and high-throughput sequencing. The original data obtained by sequencing were spliced according to the double-ended overlap region, and then low-quality sequences and chimera sequences were filtered to obtain high-quality clean data. Subsequently, sequence denoising was performed, and PCR amplification and sequencing errors in the sequencing data were removed to obtain the representative biological sequences, i.e., Amplicon Sequence Variants (ASVs), and ASV abundance data. Finally, ASV data were analyzed for α diversity, β diversity, and species composition and differences, as well as functional composition and differences.

### 2.8. Determination of the Level of SCFAs

The levels of SCFAs in fecal samples were analyzed by TSQ 9000 Gas Chromatograph (Thermo, Schaumburg, IL, USA). After adding the internal standard, the fecal samples were ground, homogenized, centrifuged, acidified and filtered, and then recorded by a gas chromatograph. The standards used were chromatographic grade acetic acid, propionic acid, butyric acid, and isobutyric acid, and the column was a JN-5MS column (30 m × 0.25 mm × 0.25 µm). The detailed computer operation steps were carried out according to the method reported by Zhai et al. [26].

### 2.9. Statistical Analysis

Analyses used a modified intention-to-treat set (all randomized with post-baseline data). The change in HAMD-17 (baseline → week 8) was tested by baseline-adjusted ANCOVA; adjusted between-group differences were shown with 95% CIs and standardized effect sizes. HAMA-14 used the same approach. Binary endpoints, response (≥50% reduction) and remission (HAMD-17 < 8; HAMA-14 < 7 at week 8), were compared by between-group logistic regression (BC99 vs. placebo, placebo as reference), adjusting for the corresponding baseline scale score, reporting adjusted odds ratios (ORs) with 95% CIs and two-sided Wald *p*-values; absolute risk differences (ARDs) with Newcombe 95% CIs were also presented. A beta–binomial sensitivity analysis with Beta (1, 1) priors estimated P (*p*_BC99_ > *p*_Placebo_) and 95% credible intervals for the risk difference. Other endpoints (PSQI, GSES; serum IL-17, IL-10, γ-GABA, NO, Cys-C, NPY; and fecal SCFAs) used baseline-adjusted ANCOVA (log-transform if skewed). 16S microbiota: α-diversity via baseline-adjusted ANCOVA; β-diversity via Bray–Curtis PERMANOVA (adonis2); and differential abundance by compositional methods with prevalence filtering. Benjamini–Hochberg FDR controlled multiplicity for secondary families. All tests were two-sided (α = 0.05). Analyses were performed in R v4.3.2. * *p* < 0.05, ** *p* < 0.01, *** *p* < 0.001, and **** *p* < 0.0001 were considered statistically significant.

## 3. Results

### 3.1. Baseline Characteristics

**Figure 2:**
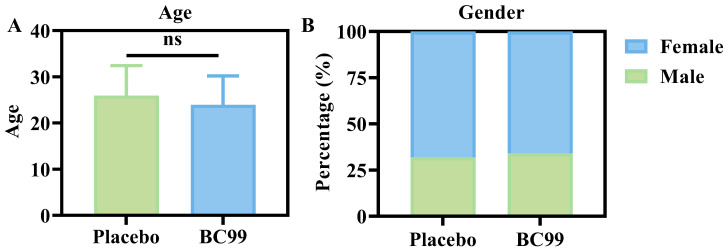
Comparison of general data between the two groups of participants; (**A**) age and (**B**) gender; ns: not signficant.

A total of 82 participants joined the 8-week clinical trial, and the baseline characteristics of these participants, including average age and gender distribution, were analyzed. As shown in Figure 2A and Table 1, there is no significant difference in gender between the BC99 group and the placebo group, with an age of 23.93 ± 6.25 years for the BC99 group and 25.94 ± 6.48 years for the placebo group. Figure 2B shows the ratio of males to females in the BC99 and placebo groups. In the placebo group (*n* = 41), 68% (*n* = 28) were females and 32% (*n* = 13) were males. In the BC99 group (*n* = 41), 66% (*n* = 27) were females and 34% (*n* = 14) were males. The differences between the BC99 group and the placebo group in age and sex were not statistically significant (*p* > 0.05). Of these, two participants (one male and one female each) dropped out of the BC99 group, and one female participant in the placebo group withdrew. In the end, only 79 people persisted until the end of the experiment. Moreover, the blood routine indicators, such as white blood cells and red blood cells, were compared between the placebo group and the BC99 group, and there was no significant difference (Table 1).

### 3.2. Comparison of HAMD and HAMA

HAMD and HAMA were compiled by Hamilton in 1960 and 1959, respectively. The CCMD-3 Diagnostic Criteria for Mental Disorders in China listed them as important diagnostic tools for mental disorders, mainly used to assess the severity of anxiety and depression symptoms in participants. According to the grade classification of HAMA-14 and HAMD-17, participants began to develop anxiety or depression symptoms when the score of the HAMA questionnaire was ≥7 points or the HAMD questionnaire score was ≥ 8 points, meaning that they met the inclusion criteria of this clinical study. The results showed that the average HAMD score of participants initially enrolled in the placebo group was 19.77, which decreased to 12.47 after an 8-week placebo intervention, while the HAMD score of the probiotic group decreased from 20.80 to 11.10 (Table 2). There was a statistically significant difference in HAMD scores between 0 and 8 weeks in the BC99 group (*p* < 0.001), indicating that BC99 could alleviate the depressive symptoms of participants. HAMA scores showed a similar trend, with a 12.5-point reduction in participants receiving probiotics, which was more than that of the placebo group. The magnitudes of baseline change in the HAMD and HAMA questionnaires were higher in the BC99 group compared with the placebo group, but there was no significant difference (*p* > 0.05).

### 3.3. Comparison of PSQI and GSES Questionnaires

**Figure 3:**
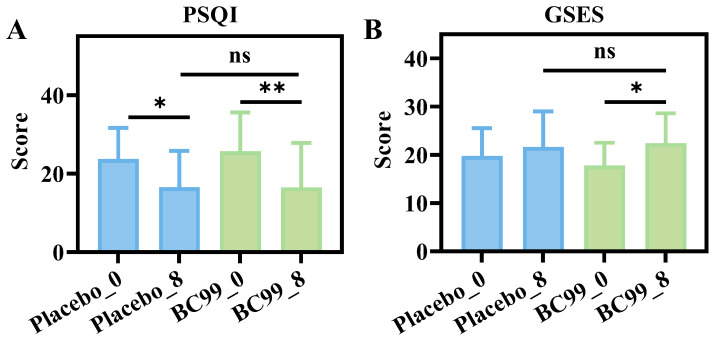
Comparison of questionnaires before and after intervention; (**A**) PSQI and (**B**) GSES; * *p* < 0.05, ** *p* < 0.01. Note: PSQI: Pittsburgh Sleep Quality Index; GSES: General Self-efficacy Scale; ns: not significant.

After 8 weeks of intervention, the PSQI score of the probiotic group was lower than that of the placebo group, and the GSES score was higher (Figure 3A,B), but there was no significant difference compared with the placebo group. This indicated that the sleep quality of participants was improved, and their self-perception, thoughts, and behaviors were also improved.

### 3.4. Inflammatory Cytokines Analysis

**Figure 4:**
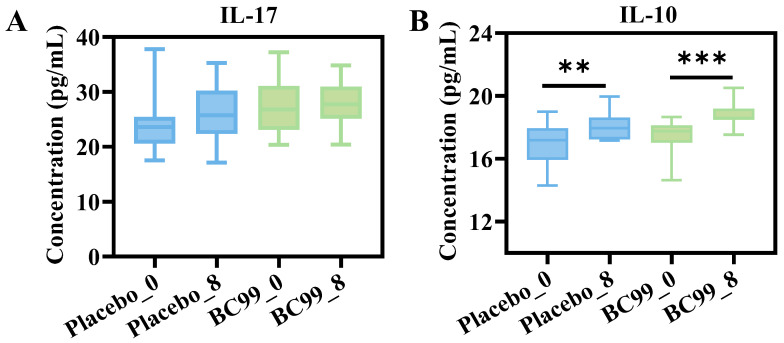
The levels of inflammatory cytokines (**A**) IL-17 and (**B**) IL-10; ** *p* < 0.01, *** *p* < 0.001. Note: IL-17: interleukin-17; IL-10: interleukin-10.

As shown in Figure 4A, the level of pro-inflammatory cytokine IL-17 increases slightly after an 8-week placebo intervention compared to baseline, whereas there is no change with the BC99 intervention. There was no significance before and after the intervention in different groups. In patients with depression, anti-inflammatory cytokine IL-10 levels are usually low, and the ratio of pro-inflammatory cytokines to anti-inflammatory cytokines increases. The experimental results showed that the IL-10 level of participants was significantly higher after an 8-week placebo intervention compared to pre-intervention (*p* < 0.01), which might be related to the placebo effect of participants (Figure 4B). In addition, IL-10 levels of the BC99 group showed a similar upward trend and were higher than those of the placebo group. This suggested that the BC99 intervention was effective in improving the levels of anti-inflammatory cytokines in participants. Lan et al. also reported that oral troxerutin could reduce the levels of TNF-α and IL-6, and increase the level of IL-10 in serum, thus alleviating the symptoms of depression by improving peripheral inflammation [27].

### 3.5. Blood Hormone Levels and Neurotransmitter Indexes Analysis

**Figure 5:**
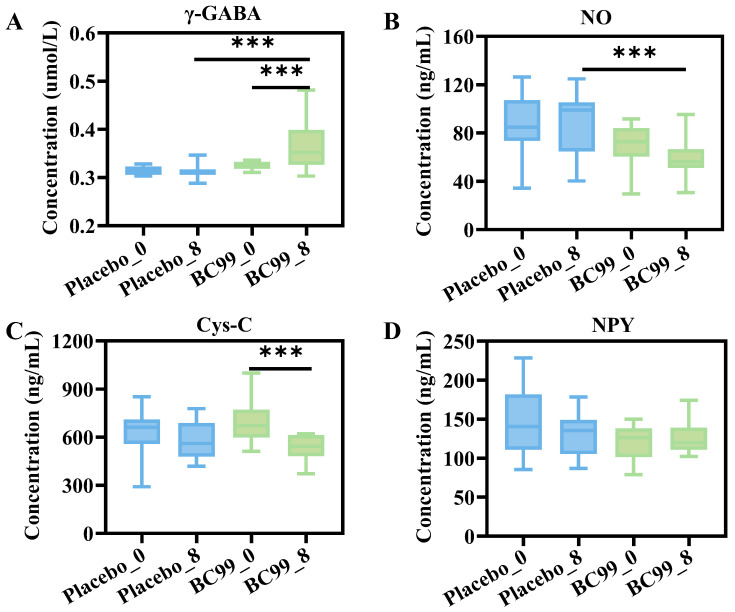
Changes in neurotransmitter levels before and after BC99 and placebo intervention. (**A**) γ-GABA, (**B**) NO, (**C**) Cys-C, and (**D**) NPY. *** *p* < 0.001. Note: γ-GABA: γ-aminobutyric acid; NO: nitric oxide; Cys-C: cystatin C; NPY: neuropeptide Y.

As shown in Figure 5A, compared with the first enrollment group, the concentration of γ-GABA in the placebo intervention group remained unchanged at 0.31 μmol/L. After 8 weeks of BC99 intervention, the level of γ-GABA increased significantly, with an increase by 0.05 μmol/L compared with the placebo group (*p* < 0.001). As a neurotransmitter, the level of NO was high in patients with major depression, and the level of NO could decrease after antidepressant treatment. As shown in Figure 5B, there is no significant difference in NO content after the placebo intervention, but the level of NO in the BC99 group is significantly lower than that in the placebo group after an 8-week intervention (*p* < 0.001). There was no significant difference in Cys-C content before and after intervention in the placebo groups (Figure 5C). After 8 weeks of BC99 intervention, the concentration of Cys-C was significantly decreased compared with week 0, from 688.90 ng/mL to 533.52 ng/mL (*p* < 0.001). Moreover, individuals with lower levels of NPY are more likely to experience negative emotional reactions, have difficulty coping with stress, and are more prone to anxiety and depression. There was no significant difference in the concentration of NPY between the placebo group and the BC99 group at week 8 (Figure 5D). To sum this up, after 8 weeks of BC99 intervention, γ-GABA level increased, and NO and Cys-C levels decreased, which might be related to the relief of anxiety and depression symptoms of the participants.

### 3.6. Effect of BC99 Intervention on Gut Microbiota

To investigate the effects of BC99 probiotic intervention on the gut microbiota of anxious and depressed participants, a 16S rRNA sequencing technique was performed on stool samples from participants. As shown in Figure S1A,B, with the increase in the sequencing number, the curves tend to be gentler, indicating that the abundance and uniformity of samples are appropriate.

**Figure 6:**
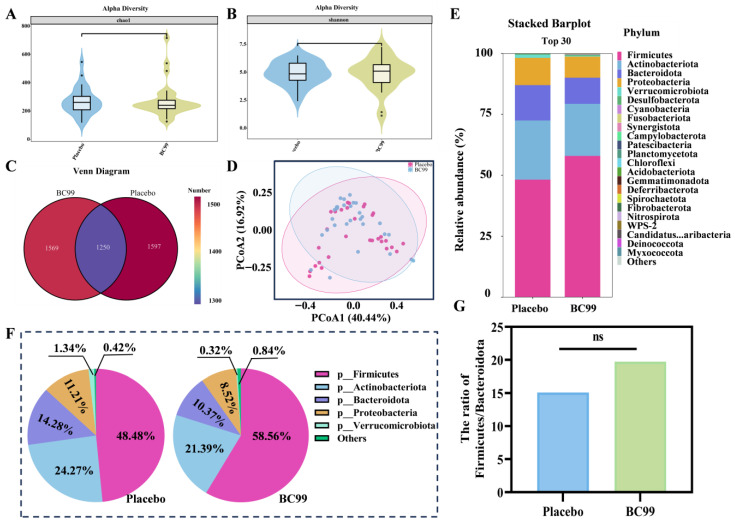
Effect of BC99 probiotic on gut microbiota in anxiety and depression participants. (**A**) Chao1 index, (**B**) Shannon index, (**C**) Veen diagram, (**D**) PCoA analysis, (**E**) Species types at phylum level, (**F**) Relative abundance content, and (**G**) The ratio of *Firmicutes* to *Bacteroidota* at phylum level; ns: not significant.

The occurrence and development of anxiety and depression are often accompanied by ecological imbalance and dysfunction of gut microbiota. Alpha and beta diversity analyses were used to evaluate the Chao1 index and Shannon index of gut microbiota in the BC99 group and placebo group at 8 weeks. As shown in Figure 6A,B, there was no significant difference in diversity between the placebo and BC99 groups. The Venn diagram also further showed that the common operational taxon unit (OTU) of the placebo and BC99 groups was 1250, of which the unique OTU of the placebo group and probiotic group were 1597 and 1569, respectively (Figure 6C). PCoA analysis showed that the confidence ellipses were not clearly separated between the two groups, but some points in the BC99 group were far apart from the placebo group, suggesting that the intervention of BC99 changed the gut microbiota structure to some extent (Figure 6D). At the phylum level, the species with relatively high abundance in the BC99 and placebo groups included *Firmicutes*, *Actinobactcriota*, *Bacteroidota*, *Proteobacteria*, and *Verrucomicrobiota*, and the proportion of these species reached more than 90% (Figure 6E,F). Compared with the placebo group, the abundance of *Actinobacteriota*, *Proteobacteria*, and *Bacteroidota* was significantly reduced, and the abundance of *Firmicutes* was increased after BC99 intervention. As beneficial bacteria, *Firmicutes* can enhance the function of the intestinal barrier by producing SCFAs, and also cross the blood–brain barrier, affect the level of neurotransmitters in the brain, and increase the expression of γ-GABA [28]. *Firmicutes* and *Bacteroidota* (F/B) play an important role in the human intestinal tract, and the F/B value can be used as a marker to evaluate intestinal health status. BC99 intervention increased the relative abundance of *Firmicutes* and F/B value compared to the placebo group (Figure 6G).

At the genus level, the differences in micro-organisms with higher abundance in the placebo and BC99 groups, such as *Bifidobacterium*, *Faecalibactrium*, *Prevotella_9*, *Megamonas*, *Dialister*, *Escherichia-Shigella*, *Agathobacter*, *Lactobacillus*, and *Eisenbergiella* were further explored (Figure S2A,B). *Faecalibactrium*, as a “mental organism”, has shown potential to intervene in depression and anxiety disorders [29]. Compared with the placebo group, the abundance of *Faecalibactrium* was significantly improved after BC99 intervention, with a 0.64-fold increase (*p* < 0.05, Figure S2B). *Prevotella_9* is a genus of bacteria that plays an important role in gut microbiota and is associated with emotional, attentional, and sensory regions in the brain [30]. However, the abundance of *Megamonas* is generally negatively correlated with anxiety degree, which means that individuals with a higher anxiety degree have a lower abundance of *Megamonas*. The abundance of *Prevotella_9* and *Megamonas* was significantly changed in the BC99 group compared with that of placebo group after 8-week intervention. *Dialister* is also the core micro-organism of gut microbiota, and the abundance of *Dialister* is negatively correlated with the anxiety and depression of participants; that is, the higher the abundance of *Dialister*, the milder the symptoms of anxiety and depression [31]. In addition, the abundance of *Agathobacter* is usually related to intestinal barrier function and immunomodulatory capacity, and its abundance may be reduced in disease states [29]. After an 8-week intervention, the levels of *Dialister* and *Agathobacter* bacteria were significantly higher in the BC99 group than those of the placebo group, with a 4.78- and 1.30-fold increase, and the difference was statistically significant (*p* < 0.05). *Escherichia-Shigella* has pro-inflammatory activity, which can exacerbate anxiety symptoms by inducing intestinal inflammation and immune responses, such as NLRP3 inflammasome activation. Studies have shown that a significant increase in the abundance of *Escherichia-Shigella* in patients with anxiety disorders is positively correlated with the severity of anxiety [30]. After an 8-week intervention, the abundance of *Escherichia-Shigella* in the BC99 group was significantly lower than the placebo group, a 0.75-fold reduction (*p* < 0.05). *Lactobacillus* can relieve anxiety symptoms by regulating neurotransmitters and receptors, and *Eisenbergiella* can also improve intestinal health by producing SCFAs. Both *Lactobacillus* and *Eisenbergiella* levels were also elevated in the BC99-treated group compared to the placebo group. This suggested that BC99 might increase the abundance of beneficial bacteria and reduce the abundance of harmful bacteria after intervention.

**Figure 7:**
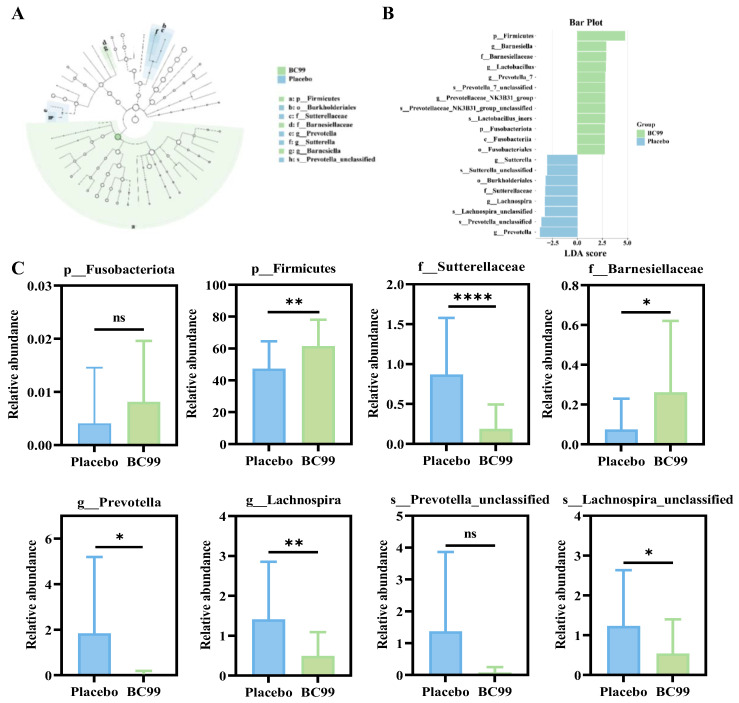
(**A**) Taxonomic cladogram of the main constituent taxa of gut microbiota. (**B**) Distribution histogram showing the significant abundance of strains, LDA score > 2.5. (**C**) The relative abundance of *Fusobacteriota*, *Firmicutes*, *Sutterellaceae*, *Barnesiellaceae*, *Prevotella*, *Lachnospira*, *Prevotella_unclassified*, and *Lachnospira_ unclassified*. * *p* < 0.05, ** *p* < 0.01, and **** *p* < 0.0001; ns: not significant.

LEfSe analysis is the commonly used microbial analysis method to identify species (biomarkers) with significant differences in abundance between different taxa by comparing placebo and BC99 groups (Figure 7A,B). LEfSe analysis is to identify the differential microbiota between the placebo group and BC99 group from different levels of community, phylum, class, order, family, genus, and species. As shown in Figure 7C, at the phylum level, the abundance of *Fusobacteriota* and *Firmicutes* increased in the BC99 group. At the family level, the abundance of *Sutterellaceae* decreased, and the abundance of *Barnesiellaceae* increased. At the genus level, the abundance of *Prevotella* and *Lachnospira* was significantly reduced in the BC99 group. At the species level, the abundance of *Prevotella_unclassified* and *Lachnospira_unclassified* also showed a downward trend.

### 3.7. SCFAs Level Analysis

**Figure 8:**
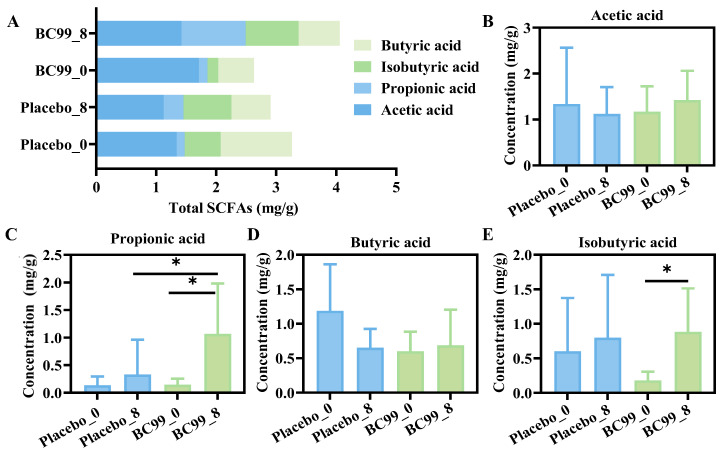
The content of SCFAs in feces. The concentration of (**A**) total SCFAs, (**B**) acetic acid, (**C**) propionic acid, (**D**) butyric acid, and (**E**) isobutyric acid. * *p* < 0.05.

As shown in Figure 8A–E, after 8 weeks of BC99 intervention, total SCFAs, acetic acid, propionic acid, butyric acid, and isobutyric acid in the BC99 group all increase to varying degrees, among which the increased degree of propionic acid and isobutyric acid is statistically significant (*p* < 0.05). Propionic acid and isobutyric acid, produced by gut microbiota, can directly affect neuronal function after crossing the blood–brain barrier, such as the release of neurotransmitters, synaptic plasticity, and neurogenesis [31]. This suggested that BC99 intervention could increase the level of SCFAs and exert health benefits in anxiety and depression participants.

### 3.8. Correlation Analysis

**Figure 9:**
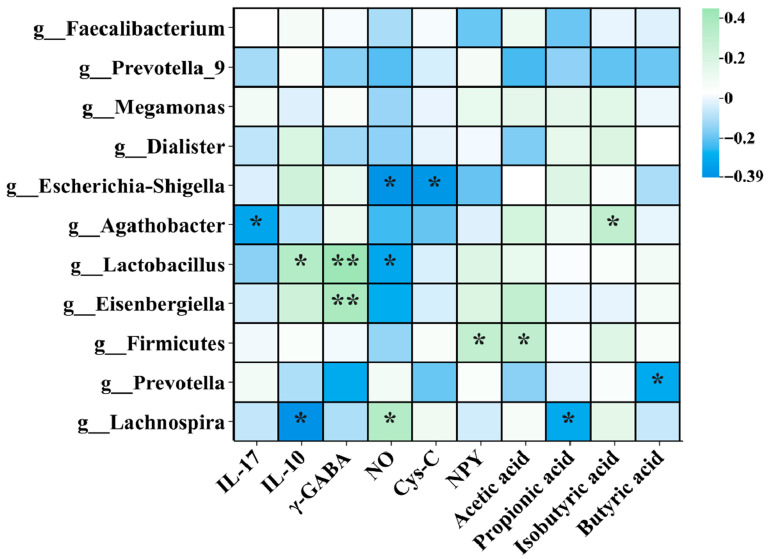
Spearman’s correlation heatmap analysis of the influence of gut microbiota. * *p* < 0.05, ** *p* < 0.01.

The potential relationship among the gut microbiota, inflammatory cytokines, neurotransmitters, and SCFAs was analyzed by Spearman’s correlation analysis. As shown in Figure 9, important bacterial genera, such as *Faecalibacterium*, *Prevotella*, *Escherichia-Shigella*, *Agathobacter*, *Lactobacillus*, *Eisenbergiella*, and *Firmicutes*, were mainly selected, and the relationship between these bacteria and metabolites was analyzed. Specifically, *Escherichia-Shigella* was negatively correlated with NO and Cys-C. *Agathobacter* was negatively correlated with IL-17, whereas positively correlated with isobutyric acid (*p* < 0.05). *Lactobacillus* had positive correlations with IL-10 and γ-GABA, and negative correlations with NO. *Eisenbergiella* had positive correlation with γ-GABA, and *Prevotella* had negative correlation with butyric acid. *Firmicutes* had a positive correlation with NPY and acetic acid (*p* < 0.05). *Lachnospira* had a positive correlation with NO, and had negative correlations with IL-10 and propionic acid (*p* < 0.05). This indicated that these strains had a strong correlation with inflammatory cytokines, neurotransmitters, and SCFAs in the body. The BC99 strain could regulate blood lipid biochemistry, neurotransmitters, and other indexes by improving the diversity and abundance of gut microbiota, thereby effectively relieving anxiety and depression.

## 4. Discussion

Anxiety and depression are common mental disorders, accompanied by persistent depression and worry, excessive or inappropriate anxiety, sleep disruption, weight change, cognitive or motor dysfunction, and other symptoms [32]. At present, the total number of people with mental disorders worldwide is close to 1 billion, and anxiety and depression affect more than 300 million and 280 million people, respectively [33,34]. The prevalence and increasing severity of these diseases have become one of the main challenges to be solved urgently in the global public health field. A high prevalence of anxiety and depression leads to an increase in socioeconomic burden, a decrease in work productivity and overall quality of life, and, in severe cases, a significant increase in the risk of developing multiple diseases such as cancer, heart disease, and suicide. However, due to the complex pathogenesis of depression, it is easily influenced by individual differences, gender, environment, and other factors, and the therapeutic effect of existing antidepressant drugs is unsatisfactory.

Experimental studies have evaluated the efficacy of probiotics in relieving depression, anxiety, and stress, such as *Bifidobacterium* and *Lactobacillus*. For example, Xie et al. studied the intervention effect of *Lactobacillus rhamnosus* KY16 (1 × 10^9^ CFU/mL) on anxiety and depression in mice, and found that oral probiotics significantly reduced stress-induced abnormal behavior and physiological dysfunction in mice after 5 weeks [15]. Nikolova et al. used compound probiotics contained 14 strains, including *Bacillus subtilis*, *Bifidobacterium bifidum*, *Bifidobacterium breve*, *Bifidobacterium infantis*, *Bifidobacterium longum*, *Lactobacillus acidophilus*, *Lactobacillus delbrueckii subsp bulgaricus*, *Lactobacillus casei*, *Lactobacillus plantarum*, *Lactobacillus rhamnosus*, *Lactobacillus helveticus*, *Lactobacillus salivarius*, *Lactococcus lactis*, and *Streptococcus thermophilus*, as the main capsule products (2 × 10^9^ CFU per capsule), and studied the therapeutic effect of 4 probiotic capsules on outpatients with severe depression and HAMD-17 score ≥ 13. The results found that after 8 weeks of probiotic intervention, the scores of HAMD-17, HAMA-14, and PSQI reduced, with high compliance and good tolerance, which provided preliminary evidence for the application of probiotics in the treatment of depression [16].

The occurrence and development of mental disorders such as anxiety and depression are closely related to neurotransmitter disorders, and the changes in the function and composition of gut microbiota play an important role in this process. Gut microbiota, as the “second brain” of the body, participates in various physiological processes such as digestion and absorption of nutrients, and immune regulation, as well as synthesis and metabolism of neurotransmitters [35]. Gut microbiota can communicate with the brain in two directions through various ways, such as nerve, endocrine, and immune ways, forming the so-called “gut microbiota-brain axis” [36]. When the balance of gut microbiota is disrupted, it may affect the synthesis and release of neurotransmitters, which in turn leads to the appearance of psychiatric symptoms such as anxiety. Based on the above analysis, the intervention of probiotics is expected to become a potentially effective way to alleviate anxiety and depression from the gut microbiota-brain axis. However, the efficacy of probiotics depends on the specific kind and dose used, as well as the underlying medical condition. *Weizmannia coagulans* BC99, a strain isolated from infant feces that survives harsh environments including high temperatures and stomach acid, has been commercialized at Wecare Probiotics Co., Ltd. Preclinical studies have shown that in the chronic sleep deprivation mouse model, BC99 supplementation could effectively alleviate anxiety/depression-like behavior and regulate gut microbiota in mice, and improve cognitive impairment by promoting SCFA production and inhibiting NLRP3 signaling pathway in the jejunum and brain [22]. This indicated that BC99 could relieve anxiety and depression by improving the gut microbiota-brain axis. This finding provides a preliminary research basis for this study to analyze the clinical efficacy of BC99 in improving anxiety/depression-related symptoms for stressed adults.

Therefore, this study analyzed the effects of BC99 (5 × 10^9^ CFU, once daily) on questionnaires, inflammatory cytokines, neurotransmitters, and gut microbiota in anxious and depressed adults. After 8 weeks of intervention, BC99 could reduce HAMD-17, HAMA-14, and PSQI questionnaire scores, and improve GSES scores. Subsequently, the HAMD and HAMA questionnaire results were further analyzed based on response rate and remission rate (Table 3). HAMD responses were 37.5% and 56.4%, and HAMD remissions were 42.5% and 56.4% in the placebo and BC99 groups, respectively. The odds ratios (OR) of response and remission were 2.41 (95% CI: 0.97–6.25) and 2.57 (95% CI: 0.94–7.48), respectively, both showing a trend towards clinical improvement for BC99, but there were no significant differences. Bayesian analysis estimated that the probability of response and remission treatment superiority of BC99 over placebo was 95.1% and 88.8%, respectively. The analysis of the HAMA questionnaire showed that the ratio of response and remission was 1.92 (95% CI: 0.76–4.96) and 1.77 (95% CI: 0.66–4.98), respectively. Placebo responses also showed an improvement trend. Kirsch et al. also studied the effects of antidepressants and placebo on depression, and found that there was no significant difference in outcomes between antidepressants and placebo, with 8.3 points and 10.1 points improvement, respectively, which was related to placebo response [37]. In addition, participants received regular and structured medical attention because they joined this pilot study believing that they were receiving treatment with beneficial effects. There were also some theories that explained that placebos were effective for all diseases, which was related to Pavlovian conditioning, resulting in no significant difference between placebo and probiotics in HAMA and HAMD outcomes.

Although the between-group differences were not statistically significant, these consistent trends toward higher response and remission rates suggested potential clinical relevance. Even moderate improvements in HAMD and HAMA scores could translate to meaningful benefits for patients, including enhanced daily functioning, reduced symptom burden, and improved quality of life. Such clinical relevance has also been observed in previous probiotic intervention studies, where probiotics showed beneficial but sometimes non-significant effects on depressive symptoms and anxiety [38,39,40]. Therefore, our findings are aligned with the existing literature, further supporting the potential of probiotics as an adjunctive strategy for mood regulation.

This result was also confirmed by inflammatory cytokine levels. IL-17 is an important pro-inflammatory cytokine mainly secreted by CD4^+^ Th17 cells, but it can also be produced by other cell types such as γδ T cells, neutrophils, and natural killer cells [41]. Many studies have shown that IL-17 may be involved in the pathogenesis of mental disorders through the following mechanisms, such as activating cells to secrete pro-inflammatory cytokines such as IL-6 and TNF-α, and destroying the blood–brain barrier and other pathways to affect the function of neurons and astrocytes, thus participating in the process of mental disorders [42,43]. IL-10 is an anti-inflammatory cytokine, mainly produced by the central nervous system, such as the pituitary gland and hypothalamus, and has a variety of important functions in the human body, especially in immune and mood regulation [44]. After BC99 intervention, the level of pro-inflammatory cytokine (IL-17) in participants was inhibited, the level of anti-inflammatory cytokine (IL-10) was increased, and the body’s inflammation was improved.

The imbalance of neurotransmitters plays an important role in the occurrence and development of anxiety and depression. γ-GABA, as an important inhibitory neurotransmitter in the brain, can reduce the excitability of neurons and maintain the balance between brain excitation and inhibition, thus producing sedative and anti-anxiety effects [45,46]. Therefore, when the level of γ-GABA was abnormal, anxiety symptoms could appear. Participants in the BC99 group had increased γ-GABA levels compared to the placebo group. NO can participate in neural signal transmission and affect many biological functions, such as learning and memory, cognitive ability, and emotional interest [47]. Moreover, NO can also affect the release of glutamate and dopamine, and participate in the neurobiological mechanism of depressive behavior. Cys-C can affect the migration of neutrophils and participate in intracellular protein transformation, collagen degradation, and protein precursor separation, thus playing an important role in the inflammatory response. This inflammatory response may aggravate depressive mood by stimulating and activating the hypothalamic–pituitary–adrenal axis, and the content of Cys-C is generally higher in patients with anxiety and depression. After the BC99 intervention, NO and Cys-C concentrations all decreased in the subject’s body. Furthermore, studies have shown that NPY plays an important role in regulating emotion, stress response, as well as anxiety and depression behavior. However, there was no significant change in NPY levels between the placebo group and the probiotic group. This indicated that BC99 might exert anti-anxiety or depressive effects by regulating serum neurotransmitter levels or inflammatory cytokines; in subsequent studies, this hypothesis could be further verified by animal or mechanism studies.

In addition, 16sRNA sequencing technology was used to analyze the changes of gut microbiota in stool samples, and the result showed that there was no significant difference in the Chao1 index and Shannon index between the placebo group and the BC99 group. Although no significant alterations in overall community structure were observed, which was consistent with some previous probiotic intervention studies in mood disorders, specific taxa-level shifts might nonetheless contribute to functional improvement through the gut–brain axis. But, compared with the placebo group, the composition of gut microbiota in the BC99-participating group changed, especially in terms of the relative abundance of specific bacterial taxa. BC99 supplementation could effectively enrich the abundance of beneficial bacteria (*Agathobacter*, *Lactobacillus*, *Eisenbergiella*, and *Firmicutes*) and inhibit the abundance of harmful bacteria (*Prevotella* and *Escherichia-Shigella*). These specific microbial types are all related to the production and regulation of inflammatory cytokines, neurotransmitters, and SCFAs. For example, *Firmicutes* could improve the function of the intestinal barrier and regulate neurotransmitter levels in the brain by producing SCFAs, and the BC99 group could effectively increase the abundance of *Firmicutes* [48]. Higher Firmicutes abundance has been associated with reduced depressive and anxiety symptoms through enhanced butyrate production, which modulates the gut-brain axis and alleviates neuroinflammation [49,50]. *Escherichia-Shigella* could induce intestinal inflammation and regulate immunity, which was positively correlated with the degree of inflammation in anxiety and depression, and was significantly reduced after BC99 intervention [51]. Decreased *Escherichia-Shigella* might improve mood by reducing pro-inflammatory cytokines (e.g., IL-1β) and enhancing gut barrier integrity, mitigating systemic inflammation linked to depression via the gut-brain axis [52,53]. Elevated *Faecalibacterium* (e.g., *F. prausnitzii*) could promote anti-inflammatory SCFAs, showing antidepressant and anxiolytic effects in animal models by modulating stress and neurotransmitters [54]. Increased *Dialister* correlated with lower depression severity, possibly via γ-GABA production and reduced neuroinflammation [55]. *Agathobacter* enrichment might stabilize mood through SCFA synthesis and gut homeostasis [56]. This indicated that BC99 supplementation could effectively regulate gut microbiota and alleviate anxiety/depression symptoms.

SCFAs are a type of fatty acid with a carbon chain length of less than six carbon atoms, of which acetic acid, propionic acid, and butyric acid account for about 90% of the total [57]. They are mainly produced by the fermentation of undigested dietary fiber and resistant starch by anaerobic bacteria in the colon. SCFAs play a variety of important roles in human health, including maintaining intestinal health, regulating the immune system, antibacterial and anti-tumor functions, and promoting energy metabolism [58,59]. They can also alleviate anxiety symptoms by affecting the production of neurotransmitters, such as serotonin and γ-GABA. These neurotransmitters play a crucial role in regulating mood and behavior, and the increase in SCFAs can promote the synthesis and function of neurotransmitters. In addition, SCFAs also have anti-inflammatory function, which can reduce systemic inflammatory responses and indirectly relieve anxiety symptoms. The level of SCFAs was significantly higher in the BC99-treated group compared to the placebo group.

In summary, BC99 supplementation increased SCFA production, likely driven by enriched *Firmicutes* and *Faecalibacterium*, which might alleviate anxiety and depression symptoms by enhancing neurotransmitter synthesis (e.g., serotonin, γ-GABA) and reducing systemic inflammation via the gut–brain axis, despite no significant changes in overall microbial diversity.

## 5. Limitations

There are still some limitations in the exploratory experiment: (1) the sample size was referred to the method reported in literatures, with 30 cases were recruited in each group, which was lower than the number recruited in this experimental study. However, the scores of HAMD-17 and HAMA-14 showed that there was no significant difference between the placebo group and the probiotic group, possibly due to the relatively small sample size, limiting the statistical power of this study. (2) The participants recruited in this study were relatively young and mainly from the same country, which limited the universality of the study results. (3) The potential intervention mechanism of BC99 on anxiety and depression through neurotransmitter or microbial changes was not further analyzed in animal models.

## 6. Conclusions

In the study, a randomized, double-blind, and placebo-controlled pilot trial was used to evaluate the clinical effect of BC99 in alleviating anxiety and depression. The preliminary results showed that both BC99 and placebo interventions were effective in reducing HAMD and HAMA questionnaire scores. The HAMD and HAMA scores in the BC99 group were reduced by 2.40 and 5.53 points compared with the placebo group, and the response and remission rates were also relatively high, but there was no significant difference compared with the placebo group. In terms of biochemical indicators, BC99 could effectively regulate the levels of inflammatory cytokines, neurotransmitters (such as γ-GABA), and SCFA. Moreover, compared with the placebo group, after 8 weeks of BC99 intervention, the abundance of beneficial bacteria closely related to anxiety and depression, such as *Faecalibactrium*, *Megamonas*, *Dialister*, *Agathobacter*, *Lactobacillus*, and *Eisenbergiella*, increased, and the abundance of harmful bacteria, *Prevotella_9* and *Escherichia-Shigella*, decreased, which could effectively regulate the gut microbiota of participants. These preliminary results could provide some scientific basis for BC99 intervention to alleviate anxiety and depression. In future clinical studies, sample size and diverse and clinically representative sample sources should be considered, and the analysis should be conducted in combination with further animal experiments to explore the effect of probiotics on anxiety and depression.

## Figures and Tables

**Table 1:** Baseline characteristics.

Projects	Placebo (*n* = 40)		BC99 (*n* = 39)		*p*-Value	Cohen’s d
	Mean	SD	Mean	SD		
Age (years)	25.94	6.48	23.93	6.25	0.1840 ^a^	0.3157
Sex (M/F)	13/27		13/26		>0.9999 ^b^	
Weight (kg)	64.22	14.07	66.92	16.87	0.4420 ^a^	−0.1738
White blood cell (10^9^/L)	5.62	1.39	5.59	0.72	0.9110 ^a^	0.0271
Red blood cells (10^12^/L)	4.42	0.57	4.38	0.31	0.7503 ^a^	0.0872
Hemoglobin (g/L)	128.18	16.63	126.82	9.10	0.7062 ^a^	0.1015
Hematocrit (%)	40.35	4.13	40.04	2.68	0.7397 ^a^	0.0891
Mean corpuscular volume (fL)	91.82	4.50	92.53	2.48	0.4696 ^a^	−0.1954
Mean corpuscular hemoglobin (pg)	29.04	0.90	29.26	0.97	0.3956 ^a^	−0.2351
Mean corpuscular hemoglobin concentration (g/L)	318.50	12.09	316.04	7.43	0.3622 ^a^	0.2452
Platelet (10^9^/L)	201.36	50.27	208.14	31.88	0.5489 ^a^	−0.1611
Lymphocyte count (10^9^/L)	1.88	0.37	1.98	0.25	0.2022 ^a^	−0.3167
Neutrophil count (10^9^/L)	3.28	1.06	3.23	0.57	0.8104 ^a^	0.0588
Lymphocyte ratio (%)	39.91	6.78	36.14	3.52	0.8768 ^a^	0.6979

Note: The statistical analysis was conducted by *t*-test ^a^, or chi-square test ^b^.

**Table 2:** The comparison of HAMD and HAMA questionnaires in the placebo and BC99 groups.

Group	Week 0	Week 8	Change from Baseline	*p*-Value	Cohen’s d
HAMD					
Placebo (*n* = 40)	19.77 ± 7.18	12.47 ± 7.84	7.30	0.0082	0.9711
BC99 (*n* = 39)	20.80 ± 9.26	11.10 ± 10.24	9.70	0.0002	0.9936
*p*-Value	0.9677	0.9295			
Cohen’s d	−0.1243	0.1502			
HAMA					
Placebo (*n* = 40)	19.80 ± 8.97	12.83 ± 9.61	6.97	0.0505	0.7498
BC99 (*n* = 39)	23.27 ± 11.94	10.77 ± 10.71	12.50	<0.0001	1.1021
*p*-Value	0.5679	0.8669			
Cohen’s d	−0.3286	0.2025			

**Table 3:** Between-group comparison of binary endpoints at week 8 (mITT).

Outcome	Placebo	BC99	OR ^a^ (95% CI)	ARD ^b^ (95% CI)	*p*-Value ^c^
HAMD Response (≥50% reduction)	37.5% (15/40)	56.4% (22/39)	2.41 (0.97–6.25)	18.9% (−11.99–46.47)	0.063
HAMD Remission (Score < 8)	42.5% (17/40)	56.4% (22/39)	2.57 (0.94–7.48)	13.9% (−16.83–42.19)	0.073
HAMA Response (≥50% reduction)	50.0% (20/40)	64.1% (25/39)	1.92 (0.76–4.96)	14.1% (−16.38–42.06)	0.17
HAMA Remission (Score < 7)	42.5% (17/40)	46.2% (18/39)	1.77 (0.66–4.98)	3.7% (−26.24–32.92)	0.268

^a^ OR from logistic regression comparing BC99 vs. placebo (reference), adjusted for baseline HAMD/HAMA score; OR > 1 favors BC99. ^b^ ARD = *P*_BC99_ − *P*_Placebo_; 95% CI by Newcombe method (Wilson score intervals then difference). ^c^ *p*-values from the logistic model (Wald test). NNT/NNH is reported only when the ARD 95% CI excludes 0; in this table, all ARD CIs cross 0, so NNT is not reported. Abbreviations: HAMD, Hamilton Depression Rating Scale; HAMA, Hamilton Anxiety Rating Scale; ARD, absolute risk difference; mITT, modified intention-to-treat.

## Data Availability

The original contributions presented in the study are included in the article; further inquiries can be directed to the corresponding authors.

## Supplementary Materials

The following supporting information can be downloaded at: https://www.mdpi.com/article/10.3390/nu17193087/s1, Figure S1: (A) species accumulation boxplot and (B) species rank curve for different treatment groups. Figure S2. (A) Relative abundance of species at genus level. (B) The relative abundance of *Faecalibactrium*, *Prevotella*, *Megamonas*, *Dialister*, *Agathobacter*, *Escherichia-Shigella*, *Lactobacillus*, and *Eisenbergiella*.
