# Supplementary material for: The Impact of Weizmannia coagulans BC99 on Anxiety and Depression: An 8-Week Clinical Pilot Study Through the Gut Microbiota–Brain Axis
Source: Nutrients. 2025 Sep 28;17(19):3087. doi: 10.3390/nu17193087 (PMC12525987; doi:10.3390/nu17193087)
Supplement: Supplementary file 1 [file nutrients-17-03087-s001.zip › nutrients-3876031-supplementary.pdf]

The Impact of *Weizmannia coagulans* BC99 on Anxiety and Depression: An 8-Week Clinical Pilot Study through the Gut Microbiota-Brain Axis

Shanshan Tie[1,2], Yujia Pan[1], Chenguang Pang[1], Saman Azeem[1], Yao Dong[3], Shuguang Fang[3], Jianguo Zhu[3], Ying Wu[1,3*], Shaobin Gu[1,2,4*]

[1] College of Food and Bioengineering, Henan University of Science and Technology, Luoyang 471000, China

[2] Henan Engineering Research Center of Food Material, Henan University of Science and Technology, Luoyang 471023, China

[3] Wecare Probiotics R&D Centers (WPC), Wecare Probiotics Co., Ltd., Suzhou 215200, China

[4] Henan Engineering Research Center of Food Microbiology, Luoyang 471000, China

* To whom correspondence should be addressed.

E-mail address:

Shaobin Gu, shaobingu@haust.edu.cn;

Ying Wu, wuying2000@126.com

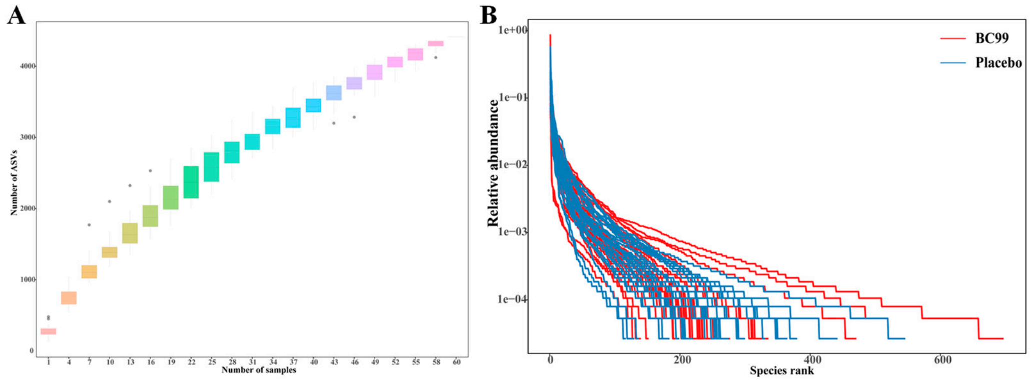

Figure S1. (A) species accumulation boxplot and (B) species rank curve for different treatment groups.

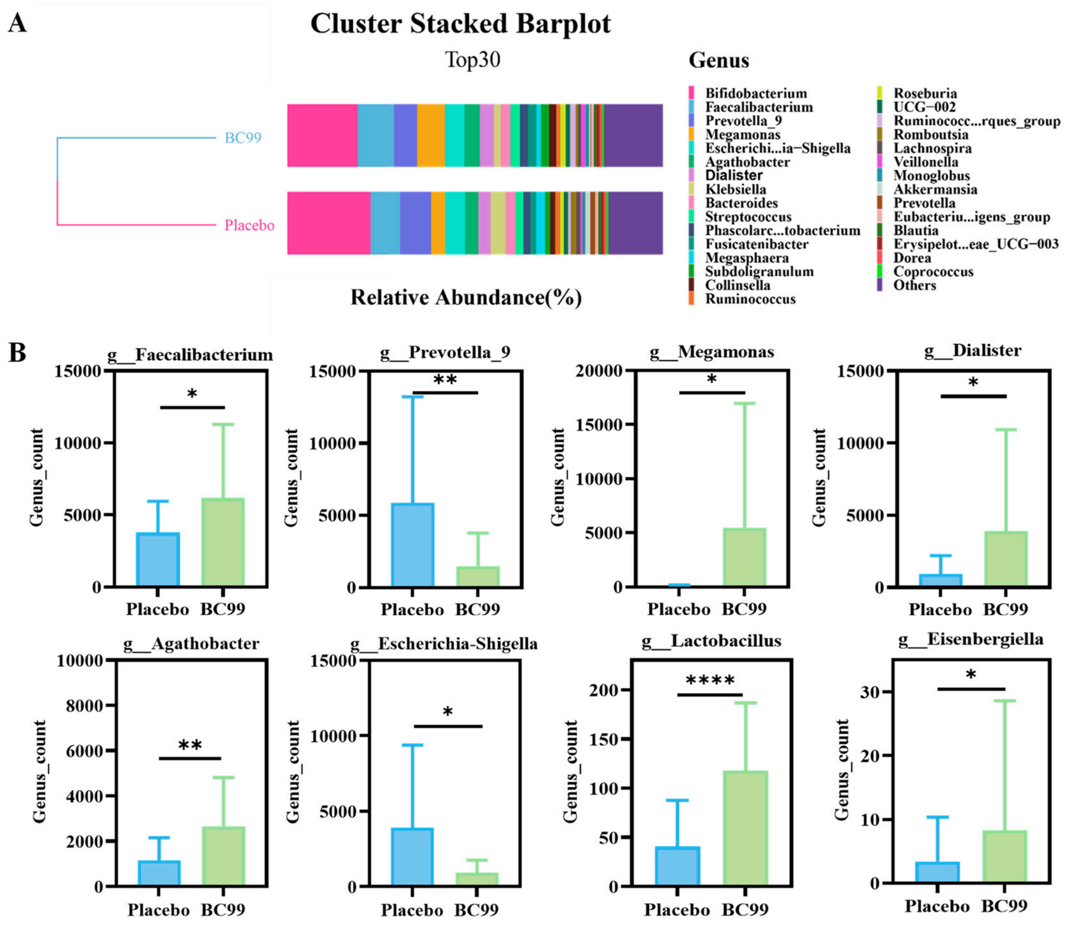

Figure S2. (A) Relative abundance of species at genus level. (B) The relative abundance of *Faecalibactrium*, *Prevotella*, *Megamonas*, *Dialister*, *Agathobacter*, *Escherichia-Shigella*, *Lactobacillus*, and *Eisenbergiella*. $*p < 0.05$, $**p < 0.01$, $***p < 0.001$, and $****p < 0.0001$.
